# The Contribution of Vessel Wall Magnetic Resonance Imaging to the Diagnosis of Primary and Secondary Central Nervous System Vasculitis

**DOI:** 10.3390/diagnostics14090927

**Published:** 2024-04-29

**Authors:** Serena D’Aniello, Arianna Rustici, Laura Ludovica Gramegna, Claudia Godi, Laura Piccolo, Mauro Gentile, Andrea Zini, Alessandro Carrozzi, Raffaele Lodi, Caterina Tonon, Massimo Dall’Olio, Luigi Simonetti, Raffaella Chieffo, Nicoletta Anzalone, Luigi Cirillo

**Affiliations:** 1 Department of Advanced Biomedical Science, University of Napoli “Federico II”, 80125 Naples, Italy; 2 Department of Biomedical and Neuromotor Sciences (DIBINEM), University of Bologna, 40138 Bologna, Italy; 3 Neuroradiology Unit, IRCCS Istituto delle Scienze Neurologiche di Bologna, Ospedale Maggiore, 40133 Bologna, Italy; 4 Vall d’Hebron Research Institute, Vall d’Hebron Barcelona Hospital Campus, 08035 Barcelona, Spain; 5 Servicio de Radiología, Unidad de Neuroradiología, Hospital del Mar, 08003 Barcelona, Spain; 6 Neuroradiology Unit and CERMAC, IRCCS San Raffaele Scientific Institute, 20132 Milan, Italy; 7 Neuroradiology Unit, Sant’Antonio Abate Hospital, ASST Valle Olona, 21013 Gallarate, Italy; 8 Neurology and Stroke Center, IRCCS Istituto delle Scienze Neurologiche di Bologna, Ospedale Maggiore, 40133 Bologna, Italy; 9 Department of Medical and Surgical Sciences (DIMEC), University of Bologna, 40138 Bologna, Italy; 10 Functional and Molecular Neuroimaging Unit, IRCCS Istituto delle Scienze Neurologiche di Bologna, 40123 Bologna, Italy; 11 Neuroradiology Unit, IRCCS Istituto delle Scienze Neurologiche di Bologna, Ospedale Bellaria, 40139 Bologna, Italy; 12 Experimental Neurophysiology Unit, IRCCS San Raffaele, Institute of Experimental Neurology (INSPE), 20132 Milan, Italy

**Keywords:** cerebral vasculitis, vessel wall, MRI, VWI, magnetic resonance

## Abstract

Background: To describe high-resolution brain vessel wall MRI (VW-MRI) patterns and morphological brain findings in central nervous system (CNS) vasculitis patients. Methods: Fourteen patients with confirmed CNS Vasculitis from two tertiary centers underwent VW-MRI using a 3T scanner. The images were reviewed by two neuroradiologists to assess vessel wall enhancement characteristics and locations. Results: Fourteen patients were included (six females; average age 48 ± 19 years). Diagnoses included primary CNS vasculitis (PCNSV) in six patients and secondary CNS vasculitis (SCNSV) in eight, half of which were infection-related. Thirteen patients showed vessel wall enhancement, which was intense in eleven patients (84.6%) and concentric in twelve (92.3%), affecting the anterior circulation in nine patients (69.2%), posterior in two patients (15.4%), and both circulations in two patients (15.4%). The enhancement patterns were similar across different CNS vasculitis types. DWI changes corresponded with areas of vessel wall enhancement in 77% of patients. Conclusions: CNS vasculitis is often associated with intense, concentric vessel wall enhancement in VW-MRI, especially in the anterior circulation. The consistent presence of DWI alterations in affected territories suggests a possible link to microembolization or hypoperfusion. These imaging findings complement parenchymal brain MRI and MRA/DSA data, potentially increasing the possibility of a clinical diagnosis of CNS vasculitis.

## 1. Introduction

Central nervous system (CNS) vasculitis is a rare, inflammatory disease affecting the medium and small intracranial vessels of the central nervous system, and is a potentially devastating cause of stroke, headache, encephalopathy, and seizure. Manifestations of cerebral vasculitis can range from neurological to psychiatric, or a combination of both, and may include cognitive decline. The multifaceted nature of its symptoms makes it a particularly challenging condition to diagnose [1,2].

CNS vasculitis can be primary (PCNS) or secondary (SCNSV) to either systemic autoimmune or infectious disorders [3,4]. Establishing the diagnosis of CNS vasculitis can be challenging due to the presence of many mimicking clinical conditions (e.g., ischemic stroke and autoimmune encephalitis) and the lack of a specific diagnostic marker [3,5]. According to the literature, PCNSVs and SCNSVs are likely to present as discrete or diffuse supra- and infratentorial ischemic white matter lesions with or without evidence of general inflammation [6].

Traditional workups include serum and cerebrospinal fluid (CSF) analyses, arterial luminal imaging with computed tomography angiography (CTA), magnetic resonance angiography (MRA), and/or digital subtraction angiography (DSA), and rarely a brain biopsy, which is still considered the gold standard for diagnoses of certainty of CNS vasculitis, even though it lacks sensitivity (samples are nondiagnostic in up to 50% of cases), and it involves an invasive procedure with the risk of serious complications [7].

No finding is specific enough to allow for a definitive diagnosis in isolation, and thus more precise neuroimaging technique capable of visualizing the vessel wall inflammatory changes can be of valuable help [2].

With the advance of neuroimaging modalities, high-resolution vessel wall magnetic resonance imaging (HR-VWI) has emerged as a non-invasive technique for the direct visualization of vessel wall inflammation and thickening in patients with intracranial arterial vasculopathies [8,9]. HR-VWI often demonstrates smooth, concentric, diffuse arterial wall thickening and strong enhancement in patients with CNS vasculitis [10,11], while eccentric wall involvement or focal lesion enhancement are all characteristics that can reliably identify vasculopathies of different origins (e.g., intracranial atherosclerotic disease—ICAD) [10,12]. Concentric vessel wall enhancement is the most frequently described feature supporting imaging diagnoses of CNS vasculitis [11]. The purpose of our study was to describe typical combinations of specific vessel wall and morphological imaging characteristics in patients with primary and secondary CNS vasculitis.

## 2. Materials and Methods

### 2.1. Study Design

This was a multicentric retrospective observational study. We conducted a retrospective review of brain MR and HR-VWI studies of patients who had received definitive clinical diagnoses of central nervous system vasculitis at the IRCCS Istituto delle Scienze Neurologiche di Bologna, Italy and IRCCS San Raffaele Scientific Institute, Italy between January 2019 and August 2021.

At each institution, patients’ neurological, clinical, and laboratory findings were reviewed by an expert neurologist who confirmed the diagnoses of vasculitis after revision of the clinical history, neurological physical examination (compatible symptoms of onset), biochemical tests (i.e., CSF exam and blood tests for autoimmune markers), and imaging studies, and after all other compatible diagnoses had been excluded [13].

All included patients had undergone at least one MR exam on a 3T scanner (MAGNETOM Skyra, Siemens Healthcare, Erlangen, Germany and Ingenia CX, Philips, The Netherlands) with 64- and 32-channel head coils including at least one parenchymal sequence (FLAIR), a diffusion-weighted sequence (DWI), a susceptibility-weighted sequence (SWI), a time-of-flight (TOF) MRA sequence, and one HR-WV-MRI sequence before and after the intravenous (i.v.) administration of paramagnetic contrast agent (Gadoteridol, Prohance^®^, Bracco, Milan, Italy 0.2 cc/Kg).

Exclusion criteria were nondiagnostic image quality and an absence of one of the mandatory imaging sequences.

The study was approved by the local ethics committee (58-2022-OSS-AUSLBO) and the need for informed consent was waived due to the retrospective nature of the study. The manuscript does not contain identifying details.

### 2.2. Imaging Assessment

HR-WVI sequences were acquired using an HR 3D T1-weighted black-blood technique before and after the i.v. administration of paramagnetic contrast agent (Gadoteridol, ProHance^®^, Bracco, Milan, Italy 0.2 cc/kg), as previously reported [14,15].

Two expert neuroradiologists, with 5 (LLG) and 15 years (LC) of experience, evaluated the images for specific signs of vasculitis through consensus. In the HR-VWI sequences, all the intracranial arteries were analyzed in detail in all three spatial planes before and after the i.v. administration of the contrast agent. Furthermore, a detailed analysis of TOF sequences with maximum intensity projection (MIP) and multi-planar reconstruction (MPR) was performed. Subsequently, images were classified as positive or negative for cerebral vasculitis based on VW-MRI features described in the literature, such as the presence of intense, concentric enhancement, most likely in multiple sites [11].

In the parenchymal FLAIR sequences, we assessed the following qualitative variables: (i) the presence of chronic white matter hyperintensities of presumed vascular origin using the three-grade modified Fazekas Scale [16]; (ii) the presence of cortical and subcortical infarcts [17]; and (iii) the age of the infarcts. In post-contrast T1-weighted images, the presence of leptomeningeal or pachymeningeal enhancement was evaluated. DWI sequences and ADC maps were analyzed for acute/subacute ischemic cortical and subcortical infarcts. SWI sequences were examined for signs of microbleeds [5,6]. In TOF sequences, the following were evaluated: (i) the presence of vessel stenosis; (ii) the presence of single or multiple stenotic vessels; (iii) the site of the main alteration (anterior or posterior circulation); and (iv) secondary sites of alterations (anterior and posterior circulation, or both). HR-VW-MRI sequences performed prior to the i.v. administration of contrast agent were scrutinized for (i) the presence of wall thickening; (ii) concentric or eccentric thickening; (iii) the site of main alteration (anterior or posterior circulation); and (iv) the presence of single or multiple thickening (i.e., in two or more vessels). Post-contrast HR-VW-MRI sequences were similarly examined for (i) the presence of wall enhancement; (ii) Ref. [18]; (iii) concentric or eccentric wall enhancement; (iv) single or multiple thickening (i.e., in two or more vessels); (v) the site of main alteration (anterior or posterior circulation); and (vi) secondary sites of alterations (anterior and posterior circulation, or both).

### 2.3. Image Evaluation

Both the readers (LLG and LC) were blinded to patients’ clinical histories and diagnoses during the evaluations, and the definitions of each item were reached by consensus.

### 2.4. Statistical Analyses

Analysis of the neurological and neuroradiological variables was performed for descriptive purposes. Descriptive statistics are reported as the mean and standard deviation (SD) for quantitative values, and count (*n*) and percentage (%) for categorical values.

## 3. Results

### 3.1. Patient Population

During the observation period, we included 14 patients (6 females). The average age was 48 ± 19 years (range: 5–83).

In total, eleven patients (78.7%) presented a symptomatic episode suggestive of stroke at the onset, one patient (7.1%) presented with a TIA, one patient (7.1%) presented with seizure, and one (7.1%) presented with headache and hearing disorders.

Six (42.9%) patients had a diagnosis of PCNSV; eight (57.1%) patients had diagnosis of SCNSV (four systemic and four infectious).

Demographics and clinical details of the included patients are displayed in Table 1.

### 3.2. Primary Central Nervous System Vasculitis

Patients’ neurological, clinical, and laboratory findings are presented in Table 2.

Parenchymal and angiographic MRI findings of PCNSV are displayed in Table 3.

### 3.3. MRI Findings

**Figure 1:**
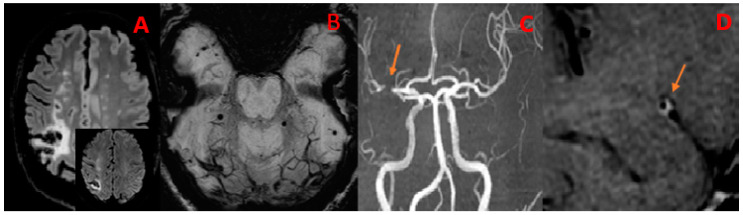
Patient (F/50) with a diagnosis of PCNSV. Ischemic stroke due to MCA M1 occlusion at the onset. (**A**) FLAIR T2-weighted image shows multiple WM alterations with ischemic lesions and a large chronic parietal infarct, with DWI hyperintensity. (**B**) SWI image shows multiple foci of bihemispheric microbleeding. (**C**) TOF MRA image shows loss of signal due to occlusion of the right M1 (arrow). (**D**) 3D SPACE T1-weighted VW image shows concentric, regular, and intense enhancement (arrow) of right M1 walls at the site of TOF alteration.

In FLAIR images, four patients (66.7%) exhibited chronic white matter lesions, presumed to be of vascular origin. Among these, all patients (100.0%) had a Fazekas score of 1. Additionally, three patients (50.0%) showed chronic cortical strokes in the middle cerebral arteries territories. The imaging of a patient with an ischemic stroke caused by M1 occlusion is depicted in Figure 1A.

In T1-weighted images obtained after the administration of the contrast agent, two (33.33%) patients presented parenchymal enhancement corresponding to the subacute ischemic lesion. None of the patients presented leptomeningeal enhancement.

In DWI images, five out of six (83.3%) patients exhibited lesions identifiable as subacute infarctions, either cortical or subcortical, within territories of both anterior and posterior circulation. All (100.0%) patients with positive DWI imaging had alterations in the territories supplied by the vessels that exhibited wall contrast enhancement in vessel wall imaging (VWI).

**Figure 2:**
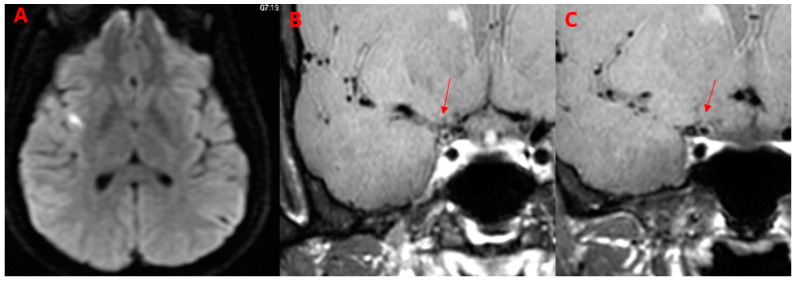
Patient (M/32) with acute onset of headache, dysarthria, and left arm weakness. The diagnosis was PCNSV. (**A**) Axial DWI image shows a right insular hyperintense area with restricted diffusion. (**B**,**C**) Coronal BB T1w VWI after administration of the contrast agent shows an intense, concentric wall enhancement (arrows) of the right sovraclinoideal internal carotid.

An example of co-present DWI and VWI alterations is displayed in Figure 2.

In SWI images, three patients (50.0%) exhibited microbleeds. The breakdown is as follows: one patient (33.3%) had hemispheric microbleeds; two (66.7%) patients showed microbleeds that were either supra- or infratentorial. Figure 1B displays the SWI results from a patient with bi-hemispheric microhemorrhages.

In TOF images, five patients (83.3%) presented arterial stenosis. Among them, two patients (40.0%) had stenosis at multiple sites (defined as two or more), and one patient (20.0%) exhibited multiple dilatations. Regarding the sites of alterations, in all five patients (100.0%), only the intracranial anterior circulation was involved (Figure 1C).

In HR-VW images before the administration of the contrast agent, four patients (66.7%) presented vessel wall thickening, of whom three (75.0%) presented thickening of a single artery and one (25.0%) presented thickening of multiple arteries. All four patients with wall thickening (100.0%) showed concentric thickening. Regarding the sites of alterations, in all four patients (100.0%), only the anterior circulation was involved.

**Figure 3:**
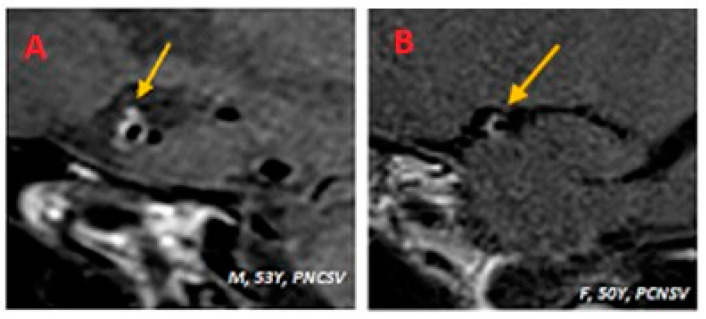
(**A**,**B**) An example of concentric and eccentric vessel wall enhancement (arrows) in two different patients with a diagnosis of PCNSV. (**A**) Patient (M/53); sagittal BB T1w VW imaging after administration of the contrast agent shows intense and concentric enhancement of the right middle cerebral artery. (**B**) Patient (F/50); sagittal BB T1w VW imaging after administration of the contrast agent shows intense and eccentric enhancement of the right middle cerebral artery.

In HR-VW-MRI images, after the administration of the contrast agent, all six patients (100.0%) presented intense enhancement. In five patients (83.3%) the enhancement was concentric, and in one patient (16.7%) it was eccentric (Figure 3). Figure 1D shows post-contrast imaging results from a patient with intense, concentric contrast enhancement of the right middle cerebral artery (M1) wall.

**Figure 4:**
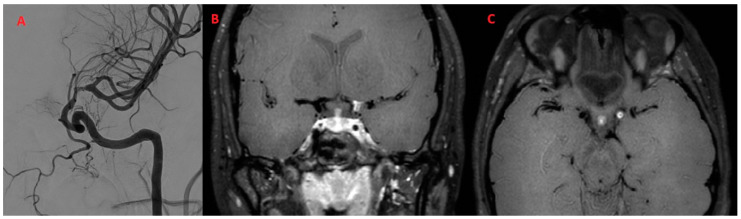
Patient (F/28) with episodes of aphasia and strength deficit of the right arm and limb with a diagnosis of PCNSV (**A**) Angiographic study shows stenosis of the distal tract of the left internal carotid. (**B**,**C**) Coronal and axial BB T1w VW imaging after administration of the contrast agent shows intense and concentric enhancement of the distal tract of the left internal carotid. No parenchymal lesions were present in FLAIR imaging.

Regarding the sites of alterations, in all patients (100.0%), only the anterior circulation was involved (Figure 4).

In post-contrast HR-VW-MRI images, three patients (50.0%) presented alterations in multiple sites.

The relationship between clinical diagnoses and imaging features in PCNSV is displayed in Table 4.

### 3.4. Secondary Central Nervous System Vasculitis

There were eight cases of SCNSV: four patients had systemic inflammatory disease with central nervous system involvement, and the other four had infectious vasculitis.

### 3.5. Secondary Central Nervous System Vasculitis—Systemic Immunomediated

There were four cases of systemic immunomediated SCNSV: one Takayasu-like syndrome, one mixed connective tissue disease, one vasculitis related to ulcerative colitis, and one giant cell arteritis.

Patients’ neurological, clinical, and laboratory findings are displayed in Table 5.

Parenchymal and angiographic MRI findings of SCNSV are displayed in Table 6.

In FLAIR images, three patients (75.0%) exhibited chronic white matter lesions, presumed to be of vascular origin. Among these, one patient (33.3%) had a Fazekas score of 1, one (33.3%) patient had a score of 2, and the remaining one (33.3%) had a score of 3. Additionally, two patients (50.0%) showed chronic cortical strokes: one (50.0%) in both the anterior and posterior circulation territories and one (50.0%) in the posterior circulation territories.

In T1-weighted images obtained after the administration of the contrast agent, two (66.7%) patients presented parenchymal enhancement corresponding to the subacute ischemic lesion; we observed leptomeningeal enhancement in no patients.

In the DWI images, two out of four patients (50.0%) exhibited lesions identifiable as subacute infarctions, either cortical or subcortical, within territories of both anterior and posterior circulation. Both patients (100.0%) showed DWI alterations in the territories supplied by the vessels that exhibited wall contrast enhancement in vessel wall imaging (VWI).

In SWI images, two patients (50.0%) exhibited microbleeds: one patient (50.0%) had hemispheric supratentorial microbleeds, and one patient (50.0%) had infratentorial microhemorrhages.

In TOF images, three patients (75.0%) presented with arterial stenosis. All of them (100.0%) had stenosis at multiple sites (defined as two or more) of both anterior and posterior circulation.

In HR-VW images before the administration of the contrast agent, two patients (50.0%) presented concentric (100.0%) vessel wall thickening.

Regarding the sites of alterations, both the anterior and posterior circulations were involved.

In HR-VW-MRI images after the administration of contrast agent, three patients (75.0%) presented concentric (100.0%), intense (100.0%) enhancement.

Regarding the sites of alterations, in one patient (33.3%) only the anterior circulation was involved, in one (33.3%) only the posterior circulation was involved, and in one (33.3%) both the anterior and posterior circulation were involved.

In 1/4 patients (25.0%) without pathological findings of vessels in TOF and HR-VW-MRI, a neuroradiological diagnosis of CNS involvement in systemic disease was suspected, observing multiple chronic infarcts on FLAIR and multiple microhemorrhages on SWI.

### 3.6. Infectious Central Nervous System Vasculitis

There were four cases of infectious SCNSV: two related to Treponema Pallidum infection (luetic), one to varicella zoster virus (VZV), and one to human immunodeficiency virus (HIV) (high replication copies positivity of Epstein–Barr virus in CSF were found, too).

Patients’ neurological, clinical, and laboratory findings are displayed in Table 7.

Parenchymal and angiographic MRI findings of SCNSV are displayed in Table 8.

**Figure 5:**
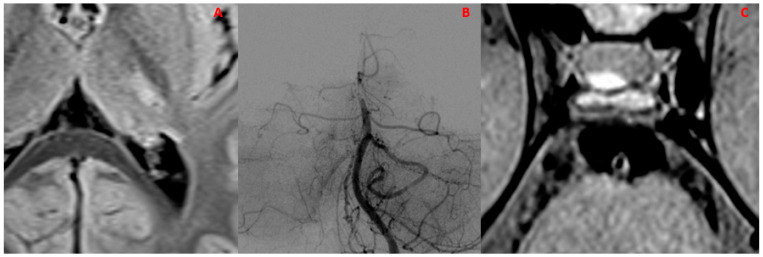
Patient (M/5) with a diagnosis of infectious (VZV-related) CNSV. (**A**) FLAIR T2-weighted image showing left thalamus hyperintensity suggestive of ischemic lesion. (**B**) Angiographic image of stenosis of the basilar artery. (**C**) 3D SPACE T1-weighted VW image shows intense, regular, concentric enhancement of walls of the basilar artery at the site of the angiographic stenosis.

In FLAIR images, three patients (75.0%) exhibited chronic white matter lesions, presumed to be of vascular origin. Among these, two patients (66.7%) had a Fazekas score of 1, and one (33.3%) patient had a score of 2. Additionally, two patients (50.0%) exhibited chronic cortical strokes: one (50.0%) in the anterior circulation territories and one (50.0%) in both the anterior and posterior circulation territories. Figure 5 showcases VWI from a patient with CNSV related to VZV.

In T1-weighted images obtained after the administration of contrast agent, two (50.0%) patients presented parenchymal enhancement corresponding to the subacute ischemic lesion; we observed leptomeningeal enhancement in no patients.

**Figure 6:**
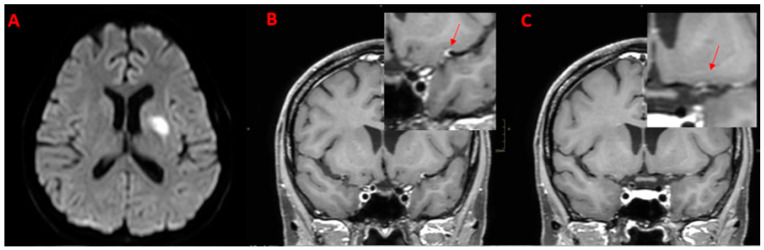
(**A**–**C**) Patient (F/39) with frequent episodes of speech impairment and arm and limb stiffness. The diagnosis was of CNS HIV-related vasculitis. (**A**) Axial DWI image shows a left nucleo-capsular hyperintense area with restricted diffusion. (**B**,**C**) Coronal BB T1w VWI after administration of the contrast agent shows an intense, concentric wall enhancement (arrow) of the left middle cerebral artery.

In the DWI images, three out of four patients (75.0%) exhibited lesions identifiable as subacute infarctions, either cortical or subcortical, within territories of both anterior and posterior circulation. All three patients (100.0%) showed DWI alterations in the territories supplied by the vessels that exhibited wall contrast enhancement in vessel wall imaging (VWI). An example of DWI alteration corresponding to territories supplied by the vessels with wall enhancement is showed in Figure 6.

In SWI images, three patients (75.0%) exhibited microbleeds: two patients (66.7%) had hemispheric infratentorial microbleeds and one patient (33.3%) had supratentorial microhemorrhages.

In TOF images, three patients (75.0%) presented with arterial stenosis. All of them (100.0%) had stenosis at multiple sites (defined as two or more) in both the anterior and posterior circulations involved.

In HR-VW images before the administration of contrast agent, three patients (75.0%) presented vessel wall thickening and all patients with wall thickening (100.0%) showed concentric thickening.

Regarding the sites of alterations, in one patient (33.3%) only the anterior circulation was involved, in one patient (33.3%) only the posterior circulation was involved, and in one (33.3%) both the anterior and posterior circulations were involved.

In HR-VW-MRI images after the administration of contrast agent, three patients (75.0%) presented intense enhancement and one (25.0%) presented mild enhancement; all four patients (100.0%) presented concentric enhancement.

**Figure 7:**
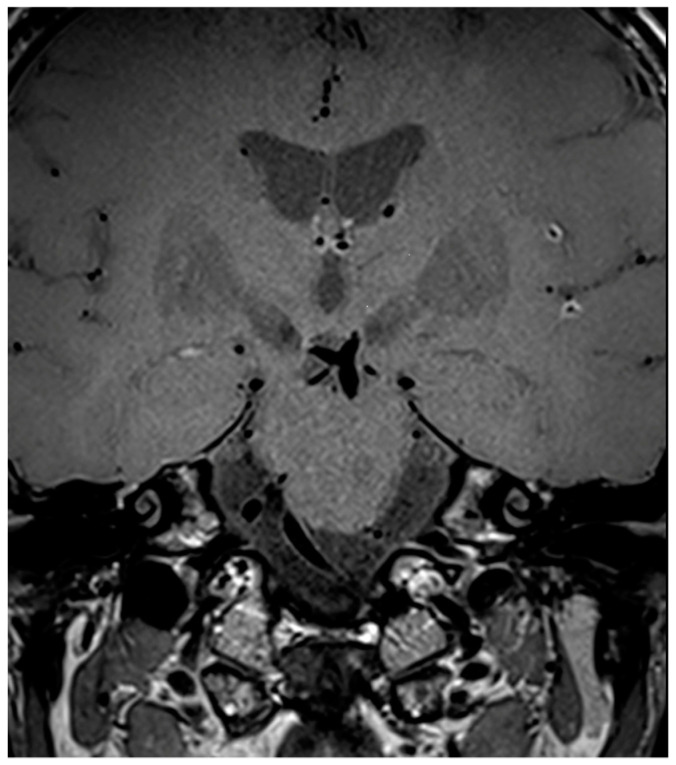
Three-dimensional SPACE T1-weighted VW image of a patient (M/49) with a diagnosis of infectious (luetic) CNSV. The image shows multiple (≥2) sites of concentric vessel wall enhancement of the distal branches of the right and left MCA.

Regarding the sites of alterations, in one patient (33.3%) only the anterior circulation was involved, in one patient (33.3%) only the posterior circulation was involved, and in one (33.3%) both the anterior and posterior circulations were involved. An instance of intense, regular, concentric contrast enhancement of the walls of the basilar artery (BA) is displayed in Figure 5. In post-contrast HR-VW-MRI images, five patients (71.4%) presented alterations in multiple sites (Figure 7).

The relationship between clinical diagnoses and imaging features in secondary immunomediated and infectious SCNSV is displayed in Table 9.

## 4. Discussion

We performed a retrospective multicenter case series of patients with a diagnosis of primary and secondary vasculitis, and we observed an overall high prevalence of intense and concentric vessel wall enhancement, most commonly in multiple arteries of the anterior circulation.

The design of our study is in line with the latest expert consensus statement (European Stroke Organization guidelines, 2023 [19]) regarding PCNSV, which considers HR-VW-MRI as a promising yet unvalidated technique, suggesting further investigation and validation through multicenter studies.

It is widely recognized that primary and secondary vasculitides involve potentially distinct pathogenic mechanisms and triggers. In primary central nervous system vasculitis (PCNSV), three histological patterns are typically observed: acute necrotizing, purely lymphocytic, and granulomatous. In secondary vasculitis, the pathophysiology may entail direct endothelial damage caused by antibodies or indirect damage from immune system mediators. Immune complex deposition in vascular walls can activate complement and coagulation cascades, thereby contributing to inflammation [20,21,22].

Secondary central nervous system (CNS) vasculitis refers to a condition where blood vessels become inflamed as a result of another underlying disease or condition. Unlike primary CNS vasculitis, secondary CNS vasculitis is associated with an underlying systemic condition, mainly referring to immunomediated and infectious diseases.

In secondary immunomediated vasculitis, the pathophysiology may entail direct endothelial damage caused by antibodies or indirect damage from immune system mediators. Immune complex deposition in vascular walls can activate complement and coagulation cascades, thereby contributing to inflammation [20,21,22]. Conversely, the pathomechanism of infectious vasculitis can vary depending on the specific infectious agent involved, but generally involves a combination of direct invasion by the pathogen and an immunomediated response. Indeed, some microorganisms have the ability to directly invade blood vessel walls, leading to inflammation and damage. This invasion can occur through various mechanisms, such as adhesion to endothelial cells lining the blood vessels, the production of toxins that damage vessel walls, or the penetration of vessel walls by the pathogen itself (e.g., varicella zoster virus and herpes simplex virus) [23]. We did not observe any cANCA- nor systemic-lupus-erythematosus-associated vasculitis. cANCA-positive forms of vasculitis are typically systemic forms affecting medium-sized vessels, although they have little predilection for the central nervous system (CNS) [24]. Systemic lupus erythematosus is a systemic autoimmune disorder that can impact multiple organs; however, CNS vasculitic involvement is uncommon [25,26]. The rarity of these conditions, combined with the small sample size of our study, likely accounts for their absence in our findings.

Nevertheless, diagnosing both primary and secondary vasculitis can be clinically challenging due to their nonspecific signs and symptoms, the difficulty in accessing CNS tissue for pathological examination, and the lack of efficient or specific non-invasive diagnostic tests.

However, inflammatory alterations of intracranial artery vessel walls can be detected by vessel wall imaging in both types of vasculitis, including the presence of concentric vivid vessel wall enhancement [27] in intracranial arteries.

In our study, the most important VW-MRI findings were concentric wall thickening in pre-contrast images (observed in all nine patients with wall thickening) and intense (100.0%) and concentric (92.3% of patients) wall enhancement in post-contrast images. These features seem to be nonspecific regarding the origin of central nervous system vasculitis, but useful for supporting the diagnosis. Multiple vessels were affected in both the anterior and posterior circulations, with a higher prevalence of middle cerebral artery involvement (9/13 patients [69.2%]). Unfortunately, our sample size was too small to perform meaningful statistical analysis. However, we observed a potentially interesting, yet subtle, difference between the involved vessels, since there was a slightly higher number of peripheral vessels (e.g., M2 distal tracts of MCA) involved in secondary immunomediated vasculitis (2/4 patients) patients compared with those with primary and secondary infectious cases, who seemed to show a more proximal (e.g., proximal tracts of MCA, ACA, VA, and BA) involvement (4/4 patients). There also seemed to be a more ubiquitous involvement of both anterior and posterior circulation in secondary vasculitides (either infectious or immunomediated) compared with the preferential (100% of our cases) involvement of the anterior circulation in PCNSV.

Diagnosing PCNSV remains challenging and involves various tools (clinical history, blood analysis, neuroradiological exams, and CSF) to exclude alternative diagnoses, particularly infectious or autoimmune diseases. In our study, patients presenting with suspected acute ischemic stroke underwent comprehensive testing to rule out systemic vasculitis or other disorders mimicking PCNSV [2]. The absence of systemic vasculitic symptoms, negative total body PET-CT, and no involvement of other organs excluded systemic vasculitis, supported by rheumatologist consultation. Although a “probable” diagnosis of PCNSV was made, a “definite” diagnosis necessitates a cerebral biopsy. Normal CSF analyses could not exclude PCNSV, but ruled out secondary vasculitis. Neuroradiological and angiographic findings, coupled with negative alternative diagnoses, heightened suspicion of cerebral vasculitis/RCVS/ICAD. RCVS was ruled out due to the non-reversibility of findings, and ICAD was deemed unlikely based on the absence of cardiovascular risk factors and response to therapy. Additionally, arterial wall thickening patterns observed were inconsistent with ICAD [10].

Regarding SCNSV, the diagnosis was established based on the positivity of microbiological examinations (e.g., HIV antibodies) or the presence of symptoms and signs of systemic disease (e.g., positive autoantibodies and PET-CT findings), including a case of reactive giant cell arteritis.

Our results confirmed the potential role of vessel wall imaging in the diagnosis of central nervous system vasculitis, as suggested by a recent review performed by Arnett N. [11], according to which the most reported VW-MRI features in case of intracranial PCNSV or SCNSV are vessel wall thickening (72%) and enhancement (89%). However, in the above-mentioned systematic review, it is reported that only 40% of the included studies further specified concentric or eccentric wall enhancement morphology. These results suggest that further research is required to provide a specific description of the type of contrast enhancement in patients with central nervous system vasculitis. In our study, consistent with previous reports [11], no differences were found between patients with infections or autoimmune origins of vasculitis. We cannot exclude that this was due to the small sample size. Nonetheless, to date, our data suggest that the presence of contrast enhancement in vessel wall imaging aids in supporting a diagnosis of central nervous system vasculitis, particularly in cases of ischemic stroke of undetermined origin, as was the situation in the majority of our cases. The presence of positive vessel wall imaging findings underscores the need for additional diagnostic analyses to determine the etiology of vasculitis.

Using TOF-MRA, we observed the presence of multiple stenotic vessels predominantly affecting the anterior circulation. There was a high concordance (12/13 [92.3%]) between alterations in TOF-MRA and HR-VW-MRI. However, in one instance, vessel wall imaging revealed abnormalities not detected as stenosis in TOF images, indicating that examining the features of the vessel walls can be beneficial, particularly when lumen-based imaging does not yield definitive results.

In our study, we only included patients with inflammation of the internal carotid arteries (ICAs), middle cerebral arteries (MCAs) (M1-M2 segments), anterior cerebral arteries (ACAs) (A1-A2 segments), intracranial vertebral arteries (VAs), basilar arteries (BAs), and the posterior cerebral arteries (PCAs) (P1-P2 segments). These segments are commonly defined as large and medium cerebral vessels in the neurological literature [28]. However, according to the Chapel Hill classification, which is the most recent international Consensus Conference categorization for all types of systemic vasculitis, including those affecting the brain, based on the size of the involved arteries (small, medium, or large vessels) [29]; these arteries would be considered medium and small-sized. In any case, vasculitis affecting the ICA, M1, M2, A1, A2, VA, BA, P1, and P2 are not amenable to biopsy, making the diagnostic process reliant on the patterns of morphological changes and vessel wall imaging along with clinical and laboratory findings.

Moreover, we excluded from our evaluation proximal tracts of intracranial ICA (2 mm) and VAs (13 mm) after dural entry, to avoid the potentially confounding concentric enhancement of vasa vasorum [30].

A feature common to all forms of cerebral vasculitis in our series was the presence of brain parenchymal changes suggestive of subacute ischemic stroke.

At the time of clinical onset, ten patients (71.4%) presented altered hyperintense lesions in DWI reflecting subacute ischemic lesions with the preferential involvement of MCA territories. Moreover, in 77.0% of patients, DWI alterations were observed in the territory of supplying vessels with wall contrast enhancement on HR-VWI. This has previously been described in the literature and suggests that vessel wall contrast enhancement represents a condition of the vessel wall that predisposes to microembolic ischemic stroke. This, in turn, might indicate that vessel wall contrast enhancement initially represents active inflammation causing prothrombogenic changes in the vessel wall and/or progressive stenosis [31].

Our study may confirm the importance of including central nervous system vasculitis in the differential diagnoses of multiple, metachronous ischemic lesions in multiple arterial territories [32,33], suggesting the occurrence of micro-embolization or hypoperfusion in association with the inflammation of the vessel wall as a pathophysiological mechanism of the patient’s symptoms. In post-contrast T1-weighted images, no patients presented meningeal enhancement. The presence of leptomeningeal enhancement has been described in up to 40% of cases in different case series [13]; however, due to the lack of post-contrast FLAIR in the acquisition, we cannot exclude by certainty the presence of this additional finding.

We also only observed parenchymal enhancements in the presence of subacute ischemic lesions (4/13 patients), indicating ruptured CSF barriers.

Furthermore, the presence of microhemorrhages in T2*-weighted/SWI imaging in CNS vasculitis (57.1% of our cases) has been largely described in the literature [34], but not in the last review on the topic [35]. Our data also suggest a nonspecific relation of CNSV with microhemorrhages, which might have been present in slightly more than half of our patients.

In conclusion, parenchymal FLAIR imaging features were aligned with the literature, showing a high prevalence of ischemic subacute lesions (75.0%).

In one case (7.1%), brain imaging aided with the diagnosis of CNS vasculitis, following negative findings on both MRA and HR-VWI; we observed multiple chronic ischemic lesions and multiple microhemorrhages on SWI.

Finally, our results align with the assertion presented in the latest guidelines from the European Stroke Organization (ESO, 2023 [19]), which emphasize that the interpretation of vessel wall imaging findings should always be conducted in conjunction with the outcomes of other morphological brain imaging modalities.

## 5. Limitations

An important limitation of this study is the small sample size, primarily attributable to the rarity of the disease under investigation. Multicenter pooling of data from large patient registers may allow for a better assessment of the value of VW-MRI as part of routine vascular MRI protocols. Additionally, histopathological analysis was not performed on any of the patients, as they all presented with large vessel vasculitis of the CNS, a condition where biopsy is deemed excessively dangerous. Furthermore, the absence of a core lab imaging review and inter-rater agreement assessment for the images constitutes a notable limitation in our analysis.

## 6. Conclusions

Vasculitis poses significant risks of permanent disability and even fatality, necessitating swift recognition and treatment. Diagnosis is complex due to diverse symptoms and a lack of standardized criteria, requiring a multidisciplinary approach involving clinical laboratory imaging and, in select cases, biopsy. However, biopsy is not always feasible and carries risks. High-resolution vessel wall imaging, although underutilized, could serve as a valuable adjunctive tool, particularly in cases of primary vasculitis where conventional diagnostics may be inconclusive. It aids in identifying vessel wall inflammation or damage, supporting the understanding of microembolization as a pathophysiological mechanism [29].

Future large-scale, multicenter studies could investigate differences in the localization of inflammatory vessels in primary and secondary CNS vasculitis. In clinical practice, radiologists are often requested to conduct vessel wall examinations for diagnosis, even prior to sufficient clinical data to distinguish between primary and secondary.

Therefore, neuroradiologists should be aware of the possible vessel wall MRI findings in patients with primary and secondary vasculitis.

## 7. Legend

Cortical Infarcts: wedge-shaped, superficial ischemic lesions in the territory of one of the large major cerebral arteries [16].

Subcortical Infarcts: small infarcts (<20 mm in diameter) located in the basal ganglia, the deep cerebral white matter, or the brainstem, and are a result of the occlusion of a single small perforating cerebral artery [16].

Chronic Infarct: no restricted diffusion on DWI was associated with the lesion.

Subacute Infarct: restricted diffusion was identified on DWI (hyperintensity in diffusion with or without hypointensity in the apparent diffusion coefficient map, depending on the age of the lesion).

Stenosis: reduction of 20% of the diameter of the vessel.

Concentric VW Thickening: >50% of wall circumference.

Eccentric VW Thickening: <50% of wall circumference.

Absent VW Enhancement: similar to adjacent vessel wall [18].

Mild VW Enhancement: lower than enhancement of the pituitary stalk but higher than the adjacent vessel wall [18].

Intense VW Enhancement: similar or higher than enhancement of the pituitary stalk [18].

Concentric VW Enhancement: >50% of wall circumference.

Eccentric VW Enhancement: <50% of wall circumference.

## Figures and Tables

**Table 1:** Demographic and clinical information.

Characteristic	*N* (%) [PCNSV, 6]	*N* (%) [Systemic SCNSV, 4]	*N* (%) [Infectious SCNSV, 4]
Gender, male	3 (50.0%)	2 (50.0%)	3 (75.0%)
Age, <50 years	2 (33.3%)	2 (50.0%)	2 (50.0%)
***Risk factors***	***PCNSV (6)***	**Systemic SCNSV (4)**	**Infectious SCNSV (4)**
Smoking	1 (16.7%)	2 (50.0%)	2 (50.0%)
Hypertension	1 (16.7%)	2 (50.0%)	1 (25.0%)
Diabetes	0 (0.0%)	3 (75.0%)	0 (0.0%)
Dyslipidemia	1 (16.7%)	3 (75.0%)	1 (25.0%)
Atrial fibrillation	0 (0.0%)	0 (0.0%)	0 (0.0%)

PCNSV, primary central nervous system vasculitis; SCNSV, secondary central nervous system vasculitis.

**Table 2:** Neurological and clinical/laboratory findings in PCNSV.

	Onset Event	CSF Test	Autoimmune Panel Tests	Other Serological Tests	PET Imaging	Clinical Diagnosis
**50Y F**	Episode suggestive of stroke	Positive (inflammatory pattern) proteins 364 (nv < 50), WBC 96 (nv < 5). Presence of oligoclonal bands	Negative	Negative	(n/a)	PCNSV
**52Y, F**	Episode suggestive of stroke	Negative	Positive (ANA 1:160, anti-gastric parietal cells 44 (nv <10)	CRP (−) ESR (+) 72 (nv < 20)	Negative	PCNSV
**53Y M**	Episode suggestive of stroke	Non-significative positive	Positive ENA+, anti-SSA RO52	Negative	Negative	PCNSV
**63Y, M**	Episode suggestive of stroke	Positive (inflammatory pattern) proteins 549 (nv < 50), WBC 171 (nv < 5). Presence of oligoclonal bands (some only intrathecal, some both in serum and in CSF in a mirror pattern)	Negative	Negative	Negative	PCNSV
**29Y, F**	Episode of TIA	Not performed (lumbar puncture contraindicated)	Negative	Negative	Negative	PCNSV
**32Y, M**	Episode suggestive of stroke	Negative	Negative	Negative	Negative	PCNSV

CSF, cerebrospinal fluid; PET, positron emission tomography; WBC, white blood cells; nv, normal value; PCNSV, primary central nervous system vasculitis; ANA, anti-nuclear antigens antibodies; CRP, C reactive protein; ESR, erythrocyte sedimentation rate; ENA, anti-extractable nuclear antigen antibodies; anti-SSA, anti-Sjögren’s-syndrome-related antigen A autoantibodies; TIA, transient ischemic attack; n/a, not available. Y = years; F = female; M = male.

**Table 3:** Parenchymal imaging findings, including DWI and SWI, and angiographical MR findings in PCNSV.

	WM Hyperintensities (Fazekas Scale)	Cortical/Subcortical Infarcts	Present/Absent DWI Hyperintensities (Subacute Events)	Site of Ischemic Lesions	Site of VWI CE	Secondary Sites of VWI CE	Present/Absent SWI Alterations (Microbleeds)	Site of SWI Alteration
**50Y, F**	1	Present	Present	Parietal lobe	MCA (M1)	None	Present	Supratentorial, subtentorial
**52Y, F**	1	Present	Present	Multiple subcortical in MCA territories	MCA M2	None	Absent	//
**53Y, M**	1	Present	Present	Fight corona radiata	ICA	ACOP	Present	Supratentorial
**63Y, M**	1	Present	Present	Multiple Subcortical in ACA, MCA Territories and Pons	ACA (A2)	None	Present	Supratentorial, subtentorial
**29Y, F**	//	Absent	Absent	No	ICA	ACA (A1), MCA (M1)	Absent	//
**32, M**	//	Absent	Present	No	ICA	ACA (A1), MCA (M1)	Absent	//

WM, white matter; DWI, diffusion-weighted imaging; VWI, vessel wall imaging; CE, contrast enhancement; SWI, susceptibility-weighted imaging; MCA, middle cerebral artery; ICA, internal carotid artery; ACOP, arteria communicans posterior; ACA, anterior cerebral artery. Y = years; F = female; M = male.

**Table 4:** Relationship between clinical diagnoses of PCNSV and imaging features.

	FLAIR CWM Hyperintensity (Fazekas Scale)	FLAIR Chronic Infarct (Cortical)	Leptomeningeal Enhancement	Parenchymal Enhancement	DWI Subacute Infarct	SWI Microbleed	MRA TOF Stenosis	Concentric VWI Wall Thickening	Eccentric VWI Wall Thickening	Concentric VWI Wall Enhancement	Eccentric VWI Wall Enhancement	Sites of CE
**PCNSV** **(*n* = 6)**	4 (66.7%)	3 (50.0%)	0 (0.0%)	2 (33.33%)	5 (83.3%)	3 (50.0%)	5 (83.3%) multiple: 2 (40.0%)	4 (66.7%)	0 (%)	5 (83.3%)	1 (16.7%)	MCA (M1, M2), ACA (A1, A2), ICA, ACOP

PCNSV, primary central nervous system vasculitis; FLAIR, fluid-attenuated inversion recovery; CWM, cerebral white matter; DWI diffusion-weighted imaging; SWI, susceptibility-weighted imaging; MRA, magnetic resonance angiography; TOF, time-of-flight; VWI, vessel wall imaging; CE, contrast enhancement; MCA, middle cerebral artery; ICA, internal carotid artery; ACOP, arteria communicans posterior; ACA, anterior cerebral artery.

**Table 5:** Neurological and clinical/laboratory findings in systemic inflammatory SCNSV.

	Onset Event	CSF Test	Autoimmune Panel Tests	Other Serological Tests	PET Imaging	Clinical Diagnosis
**66Y, M**	Episode suggestive of stroke	Negative	Positive ANA + (low title)	PCR (+)	Large-vessel uptake (aortic-supra-aortic vessels)	Systemic CNSV (Takayasu-like syndrome)
**32Y, F**	Seizures	Positive (inflammatory pattern) proteins 141 (nv < 50), WBC 9 (nv < 5),	Positive ANA 1:320, ENA + (SSA, anti-RNP)	CRP (+) ESR (+) 106 (nv < 20)	(n/a)	Systemic CNSV (mixed connective tissue disease)
**49Y, M**	Episode suggestive of stroke	Negative	Positive Anti-ENA (+)	(n/a)	Negative	Systemic CNSV (ulcerative colitis + vasculitis)
**83Y, F**	Episode suggestive of stroke	(n/a)	Negative	CRP (+) ESR (+) 46 (nv < 20)	Negative	Systemic CNSV (GCA flare-up with CNS involvement)

CSF, cerebrospinal fluid; PET, positron emission tomography; CNSV, central nervous system vasculitis; nv, normal value; ANA, anti-nuclear antigens antibodies; CRP, C reactive protein; ESR, Erythrocyte sedimentation rate; ENA, anti-extractable nuclear antigen antibodies; anti-SSA, anti-Sjögren’s-syndrome-related antigen A autoantibodies; WBC, white blood cells; GCA, giant cell arteritis; n/a, not available, Y = Years, M = Male, F = Female.

**Table 6:** Parenchymal findings, including DWI and SWI, and angiographical MR findings in systemic inflammatory SCNVS.

	WM Hyperintensities (Fazekas Scale)	Cortical/Subcortical Infarcts	Present/Absent DWI Hyperintensities (Subacute Events)	Site of Ischemic Lesions	Site of VWI CE	Secondary Sites of VWI CE	Present/Absent SWI Alterations (Microbleeds)	Site of SWI Alteration
**66Y, M**	3	Present	Present	Bilateral Basal Ganglia, Left SCA	SCA	ACM (M1) ICA BA	Present	Subtentorial
**32Y, F**	1	Absent	Absent	No	None	None	Absent	//
**49Y, M**	//	Absent	Absent	No	ACM (M2)	ACM (M2)	Present	Supratentorial
**83Y, F**	2	Present	Present	Cortical-Subcortical in PCA Territory	PCA (P2)	None	Absent	//

WM, white matter; DWI, diffusion-weighted imaging; VWI, vessel wall imaging; CE, contrast enhancement; SWI, susceptibility-weighted imaging; ICA, internal carotid artery; SCA, superior cerebellar artery; BA, basilar artery; PCA, posterior cerebral artery.

**Table 7:** Neurological and clinical/laboratory findings in infectious SCNSV.

	Onset Event	CSF Test	Autoimmune Panel Tests	Other Serological Tests	PET Imaging	Clinical Diagnosis
**49y, M**	Headache, hearing disorders	Positive (inflammatory pattern + T. Pallidum positivity) proteins 82 (nv < 50), WBC 73 (nv < 5), TPHA liquor + 1:640	Negative	T. Pallidum antibody-positive (TPHA > 1:640, RPR +)	(n/a)	Luetic CNSV
**58Y, M**	Episode suggestive of stroke	Positive (inflammatory pattern + T. Pallidum positivity) proteins 103 (nv < 50), WBC 17 (nv < 5), TPHA liquor + 1:640	Negative	CRP (+), T. Pallidum antibody-positive CRP 1,7 (nv < 0.5), ESR 52 (nv < 20), T. Pallidum antibody-positive (TPHA 1:640, RPR +)	Large-vessel uptake	Luetic CNSV
**5Y, M**	Episode suggestive of stroke	(n/a)	Negative	VZV-IgM antibody-positive CRP (+)	(n/a)	VZV-CNSV
**39Y, F**	Episode suggestive of stroke	Positive (inflammatory pattern + HIV antibodies + Epstein–Barr virus positivity 490 copies/mL)	Negative	CRP (+)	(n/a)	HIV/EBV-related CNSV

CSF, cerebrospinal fluid; PET, positron emission tomography; CNSV, central nervous system vasculitis; nv, normal value; CRP, C reactive protein; ESR, erythrocyte sedimentation rate; TPHA, treponema pallidum hemagglutination; VZV, varicella zoster virus; HIV, human immunodeficiency virus; EBV Epstein–Barr virus. Y = Years, M = Male, F = Female.

**Table 8:** Parenchymal findings, including DWI and SWI, and angiographical MR findings in infectious SCNVS.

	WM Hyperintensities (Fazekas Scale)	Cortical/Subcortical Infarcts	Present/Absent DWI Hyperintensities (Subacute Events)	Site of Ischemic Lesions	Site of VWI CE	Secondary Sites of VWI CE	Present/Absent SWI Alterations (Microbleeds)	Site of SWI Alteration
**49Y, M**	1	Present	Present	Multiple subcortical in MCA territories	ICA	MCA (M1) ACA (A1)	Present	Subtentorial
**58Y, M**	2	Present	Present	Multiple subcortical in ACA and MCA territories and left SCA	ICA	None	Present	Supratentorial
**5Y, M**	//	Absent	Absent	No	VA	BA	Present	Subtentorial
**39Y, F**	1	Absent	Present	No	M1	PCA (P1)	Absent	//

WM, white matter; DWI, diffusion-weighted imaging; VWI, vessel wall imaging; CE, contrast enhancement; SWI, susceptibility-weighted imaging; MCA, middle cerebral artery; ICA, internal carotid artery; SCA, superior cerebellar artery; VA, vertebral artery; BA, basilar artery; ACA, anterior cerebral artery; PCA, posterior cerebral artery. Y = Years, M = Male, F = Female.

**Table 9:** Relationship between clinical diagnoses of both systemic (upper table) and infectious (lower table) SCNSV and imaging features.

	FLAIR CWM Hyperintensity (Fazekas Scale)	FLAIR Chronic Infarct (Cortical)	Leptomeningeal Enhancement	Parenchymal Enhancement	DWI Subacute Infarct	SWI Microbleed	MRA TOF Stenosis	Concentric VWI Wall Thickening	Eccentric VWI Wall Thickening	Concentric VWI Wall Enhancement	Eccentric VWI Wall Enhancement	Sites of VWI CE
**SCNSV Systemic disease (*n* = 4)**	3 (75.0%)	2 (50.0%)	0 (0.0%)	2 (50.0%)	2 (50.0%)	2 (50.0%)	3 (75.0%)	2 (50.0%)	0 (0%)	3 (75.0%)	0 (0%)	BA, VA, SCA, ICA, ACM (M1), PCA (P2)
							multiple: 3 (100.0%)					
	**FLAIR CWM Hyperintensity (Fazekas Scale)**	**FLAIR Chronic Infarct (Cortical)**	**Leptomeningeal Enhancement**	**Parenchymal Enhancement**	**DWI subacute Infarct**	**SWI Microbleed**	**MRA TOF** **Stenosis**	**Concentric VWI Wall Thickening**	**Eccentric VWI Wall Thickening**	**Concentric VWI Wall Enhancement**	**Eccentric VWI Wall Enhancement**	**Sites of VWI CE**
**SCNSV Luetic (*n* = 2)**	2 (100.0%)	2 (100.0%)	0 (0.0%)	0 (0.0%)	2 (100.0%)	2 (100.0%)	1 (50.0%) multiple: 1 (100.0%)	1 (50.0%)	0 (0%)	2 (100.0%)	0 (0%)	ACM(M1), ACA(A1), ICA
**SCNSV VZV-related (*n* = 1)**	0 (0.0%)	0 (0.0%)	0 (0.0%)	0 (0.0%)	0 (0.0%)	1 (100.0%)	1 (100.0%)	1 (100.0%)	0 (0%)	1 (100.0%)	0 (0%)	BA, VA
							multiple: 1 (100.0%)					
**SCNSV HIV/EBV-related (*n* = 1)**	1 (100.0%)	0 (0.0%)	0 (0.0%)	0 (0.0%)	1 (100.0%)	0 (0.0%)	1 (100.0%) multiple: 1 (100.0%)	1 (100.0%)	0 (0.0%)	1 (100.0%)	0 (0.0%)	ICA, ACA (A1), MCA (M1)

SCNSV, primary central nervous system vasculitis; FLAIR, fluid-attenuated inversion recovery; CWM, cerebral white matter; DWI diffusion-weighted imaging; SWI, susceptibility-weighted imaging; MRA, magnetic resonance angiography; TOF, time-of-flight; VWI, vessel wall imaging; CE, contrast enhancement; MCA, middle cerebral artery; ICA, internal carotid artery; SCA, superior cerebellar artery; VA, vertebral artery; BA, basilar artery; ACA, anterior cerebral artery; PCA, posterior cerebral artery.

## Data Availability

Data are available upon reasonable request. Requests should be made to the corresponding author.

## References

[1] Salvarani C., Brown R.D., Hunder G.G. (2012). Adult primary central nervous system vasculitis. Lancet.

[2] Calabrese L.H., Mallek J.A. (1988). Primary angiitis of the central nervous system. Report of 8 new cases, review of the literature, and proposal for diagnostic criteria. Medicine.

[3] Hajj-Ali R.A., Calabrese L.H. (2020). Central nervous system vasculitis: Advances in diagnosis. Curr. Opin. Rheumatol..

[4] Godasi R., Pang G., Chauhan S., Bollu P.C. (2024). Primary Central Nervous System Vasculitis. StatPearls.

[5] Ferro F., Quartuccio L., Monti S., Delvino P., Di Cianni F., Fonzetti S., La Rocca G., Posarelli C., Treppo E., Talarico R. (2021). One year in review 2021: Systemic vasculitis. Clin. Exp. Rheumatol..

[6] Abdel Razek A.A., Alvarez H., Bagg S., Refaat S., Castillo M. (2014). Imaging spectrum of CNS vasculitis. Radiographics.

[7] Néel A., Pagnoux C. (2009). Primary angiitis of the central nervous system. Clin. Exp. Rheumatol..

[8] Merli E., Rustici A., Gramegna L.L., Di Donato M., Agati R., Tonon C., Lodi R., Favoni V., Pierangeli G., Cortelli P. (2023). Vessel-wall MRI in primary headaches: The role of neurogenic inflammation. Headache.

[9] Rustici A., Merli E., Cevoli S., Donato M.D., Pierangeli G., Favoni V., Bortolotti C., Sturiale C., Cortelli P., Cirillo L. (2021). Vessel-wall MRI in thunderclap headache: A useful tool to answer the riddle?. Interv. Neuroradiol..

[10] Mossa-Basha M., Hwang W.D., De Havenon A., Hippe D., Balu N., Becker K.J., Tirschwell D.T., Hatsukami T., Anzai Y., Yuan C. (2015). Multicontrast high-resolution vessel wall magnetic resonance imaging and its value in differentiating intracranial vasculopathic processes. Stroke.

[11] Arnett N., Pavlou A., Burke M.P., Cucchiara B.L., Rhee R.L., Song J.W. (2022). Vessel wall MR imaging of central nervous system vasculitis: A systematic review. Neuroradiology.

[12] Wu F., Ma Q., Song H., Guo X., Diniz M.A., Song S.S., Gonzalez N.R., Bi X., Ji X., Li D. (2018). Differential Features of Culprit Intracranial Atherosclerotic Lesions: A Whole-Brain Vessel Wall Imaging Study in Patients With Acute Ischemic Stroke. J. Am. Heart Assoc..

[13] Berlit P., Kraemer M. (2014). Cerebral vasculitis in adults: What are the steps in order to establish the diagnosis? Red flags and pitfalls. Clin. Exp. Immunol..

[14] Cirillo L., Rustici A., Toni F., Zoli M., Bartiromo F., Gramegna L.L., Cicala D., Tonon C., Caranci F., Lodi R. (2022). Vessel Wall MRI: Clinical implementation in cerebrovascular disorders-technical aspects. Radiol. Med..

[15] Godi C., Destro F., Garofalo P., Tombetti E., Ambrosi A., Iadanza A., Michelozzi C., Falini A., Anzalone N. (2023). Hemodynamic nature of black-blood enhancement in long-term coiled cerebral aneurysms. Neuroradiology.

[16] Fazekas F., Chawluk J.B., Alavi A., Hurtig H.I., Zimmerman R.A. (1987). MR signal abnormalities at 1.5 T in Alzheimer’s dementia and normal aging. AJR Am. J. Roentgenol..

[17] Venkataraman P., Tadi P., Lui F. Lacunar Syndromes. https://www.ncbi.nlm.nih.gov/books/NBK534206/.

[18] Mandell D.M., Mossa-Basha M., Qiao Y., Hess C.P., Hui F., Matouk C., Johnson M.H., Daemen M.J., Vossough A., Edjlali M. (2017). Intracranial Vessel Wall MRI: Principles and Expert Consensus Recommendations of the American Society of Neuroradiology. AJNR Am. J. Neuroradiol..

[19] Pascarella R., Antonenko K., Boulouis G., De Boysson H., Giannini C., Heldner M.R., Kargiotis O., Nguyen T.N., Rice C.M., Salvarani C. (2023). European Stroke Organisation (ESO) guidelines on Primary Angiitis of the Central Nervous System (PACNS). Eur. Stroke J..

[20] Miller D.V., Salvarani C., Hunder G.G., Brown R.D., Parisi J.E., Christianson T.J., Giannini C. (2009). Biopsy findings in primary angiitis of the central nervous system. Am. J. Surg. Pathol..

[21] Salvarani C., Brown R.D., Hunder G.G. (2012). Adult primary central nervous system vasculitis: An update. Curr. Opin. Rheumatol..

[22] Harland T.A., Seinfeld J., Cava L.F., Neumann R.T., Roark C., Kumpe D., Case D. (2019). Anti-neutrophil cytoplasmic antibody associated central nervous system vasculitis with brain and spinal cord subarachnoid hemorrhage: A rare case report and review of the literature. J. Clin. Neurosci..

[23] Pipitone N., Salvarani C. (2008). The role of infectious agents in the pathogenesis of vasculitis. Best. Pract. Res. Clin. Rheumatol..

[24] Graf J. (2017). Central Nervous System Disease in Antineutrophil Cytoplasmic Antibodies-Associated Vasculitis. Rheum. Dis. Clin. N. Am..

[25] Rodrigues M., Galego O., Costa C., Jesus D., Carvalho P., Santiago M., Malcata A., Ines L. (2017). Central nervous system vasculitis in systemic lupus erythematosus: A case series report in a tertiary referral centre. Lupus.

[26] Rowshani A.T., Remans P., Rozemuller A., Tak P.P. (2005). Cerebral vasculitis as a primary manifestation of systemic lupus erythematosus. Ann. Rheum. Dis..

[27] Mazzacane F., Mazzoleni V., Scola E., Mancini S., Lombardo I., Busto G., Rognone E., Pichiecchio A., Padovani A., Morotti A. (2022). Vessel Wall Magnetic Resonance Imaging in Cerebrovascular Diseases. Diagnostics.

[28] Küker W. (2007). Imaging of cerebral vasculitis. Int. J. Stroke.

[29] Jennette J.C., Falk R.J., Bacon P.A., Basu N., Cid M.C., Ferrario F., Flores-Suarez L.F., Gross W.L., Guillevin L., Hagen E.C. (2013). 2012 revised International Chapel Hill Consensus Conference Nomenclature of Vasculitides. Arthritis Rheum.

[30] Guggenberger K.V., Torre G.D., Ludwig U., Vogel P., Weng A.M., Vogt M.L., Fröhlich M., Schmalzing M., Raithel E., Forman C. (2022). Vasa vasorum of proximal cerebral arteries after dural crossing–potential imaging confounder in diagnosing intracranial vasculitis in elderly subjects on black-blood MRI. Eur. Radiol..

[31] Birnbaum J., Hellmann D.B. (2009). Primary angiitis of the central nervous system. Arch. Neurol..

[32] Mossa-Basha M., Alexander M., Gaddikeri S., Yuan C., Gandhi D. (2016). Vessel wall imaging for intracranial vascular disease evaluation. J. Neurointerv. Surg..

[33] Edjlali M., Qiao Y., Boulouis G., Menjot N., Saba L., Wasserman B.A., Romero J.M. (2020). Vessel wall MR imaging for the detection of intracranial inflammatory vasculopathies. Cardiovasc. Diagn. Ther..

[34] Blitstein M.K., Tung G.A. (2007). MRI of cerebral microhemorrhages. AJR Am. J. Roentgenol..

[35] Haller S., Vernooij M.W., Kuijer J.P.A., Larsson E.M., Jäger H.R., Barkhof F. (2018). Cerebral Microbleeds: Imaging and Clinical Significance. Radiology.

